# Prevalence of *Brucella *species in unpasteurized dairy products consumed in Shiraz province using PCR assay

**DOI:** 10.22099/mbrc.2020.37381.1506

**Published:** 2020-09

**Authors:** Fargol Abdali, Saeid Hosseinzadeh, Enayat Berizi, Maryam Pourmontaseri

**Affiliations:** 1 Nutrition Research Center, Department of Food Hygiene and Quality Control, School of Nutrition and Food Sciences, Shiraz University of Medical Sciences, Shiraz, Iran; 2 Department of Food Hygiene and Public Health, School of Veterinary Medicine, Shiraz University, Shiraz, Iran

**Keywords:** Consumption, Brucella, Dairy products, PCR

## Abstract

The consumption of milk and unpasteurized dairy products contaminated with Brucella bacteria is one of the most important ways of brucellosis transmission to humans. The principal goal of this study was to determine the prevalence of *Brucella abortus* (*B. abortus*) and *Brucella melitens *(*B. melitens*) in unpasteurized dairy products consumed in Shiraz province. In this study conducted in 2016, 238 unpasteurized dairy products including 48 raw milk, 48 yogurt, 46 cheeses, 48 dough and 48 ice cream samples, were purchased from the retail market in Shiraz province and were examined by a specific PCR assay. This study showed positive 5/04% out of 238 unpasteurized dairy products including 9 out of 48 (18/75%) raw milk samples and 3 out of 48 (6.25%) yogurt samples). Contamination was not detected in samples of dough, cheese and traditional ice cream. The results also showed that among 12 positive samples, 6 samples were contaminated with *B. abortus* (including 4 milk samples and 2 yogurt samples), 2 samples were contaminated with *B. melitensis* (including 2 Milk samples) and 4 samples were contaminated simultaneously with *B. abortus* and *B. melitensis* (including 3 milk samples and 1 yogurt sample). The present study suggests the unpasteurized dairy products as the major sources of brucellosis in Shiraz province, South of Iran; thus, to prevent brucellosis in human, the consumption of pasteurized milk and dairy products is highly recommended.

## INTRODUCTION

Brucellosis is considered as one of the important zoonotic infections affecting both humans and animals [1]. Brucella are gram negative facultative intracellular coccobacilli that lack capsules, flagella, and endospores [2]. Ten main species have been already described which are classified as the major pathogenic species, worldwide, responsible for bovine brucellosis and *B. melitensis* is acknowledge as the main causative agent of ovine and caprine brucellosis, while swine brucellosis is caused by *B. suis*. However, few important consequences including abortion and retained placenta are caused by these three species of *Brucella* involved in massive economic losses in the endemic areas including Iran [3]. *B. melitensis* is more important than any other species of *Brucella* due to its severe pathogenicity in human [4].

In terms of the number of people affected with brucellosis in the human populations of the countries of the region, the highest incidence is related to the countries such as Saudi Arabia, Iran, Palestine, Syria, Jordan and Oman [5].Brucellosis a serious public health issue in our country. *B. melitensis* was firstly isolated from human blood culture in 1932. The first vaccination program of cattle population was employed as early as 1949 [6]. This bacterium enters the body through the mucous membrane and through the bloodstream reaches organs such as the liver, spleen, bone marrow and kidneys and ultimately causing illness [7]. The onset of the disease is gradual and involves cough, fever, weakness, pain and sweating. Gastrointestinal and nervous signs may also be present. It also causes various complications in humans such as urinary tract obstruction, liver problems such as hepatitis, acute renal failure, pericarditis, and inflammation of the joints [8]. Today, manufacturing milk and its products are one of the major trading activities; even in some countries, per capita consumption of milk in the community has been mentioned as an indicator of progress and development. Due to the high nutritional standards of milk and other dairy products, they are playing increasingly important roles in human nutrition. On the other hand, because of high nutritional value, it is a decent environment for the proper growth and activity of different pathogenic microorganisms [9]. Therefore, many health problems are threatening the consumers of such food stuffs. Brucellosis is one of the most dangerous infectious diseases that is transmitted to humans via the ingestion of contaminated milk and other dairy products. Since the agent of disease is excreted through the secretions in the milk of infected livestock, the consumption of milk and unpasteurized dairy products in the Brucella-infected areas is one of the main routes for transmitting brucellosis to humans [10].

It is necessary to employ the current established diagnostic methods leading to accurate diagnosis of Brucella and decreasing the possibility of infection with the bacterium in the laboratory. The highly sensitive PCR assay has successfully employed to detect the acute and chronic human brucellosis and is more specific than serological testing. In addition, working on DNA reduces the risk of the laboratory infection caused by cultivation [11, 12].

Considering the important consequences of the infection and limited available information on its prevalence in southern Iran, the present study was conducted to determine the contamination rates of *B. melitensis* and *B. abortus* in milk and unpasteurized dairy products consumed in Shiraz province using a PCR assay.

## MATERIALS AND METHODS

**Sample collection: **This cross-sectional descriptive study was conducted from summer 2016 to winter 2016 on the total of 238 unpasteurized dairy products samples, (48 raw milk, 48 yoghurt, 46 cheeses, 48 dough and 48 ice cream samples). The samples were randomly prepared from the retail market in Shiraz province and transported to the laboratory in the sterilized containers on ice which were stored at -20°C until further use.

**DNA extraction: **DNA extraction was performed using DNA extraction kit (Cinna Gene, Iran). The bacterial preparations were initially transferred into 1.5 μL microfuge tubes and centrifuged for 10 minutes at 12000 rpm. The supernatant was removed and then 200 μL of lysis buffer and 40 μL of proteinase K were added. Then, 30 μl of DNase free water was added to the DNA precipitate and kept at -20°C until further use. The quantity of DNA was recorded using ANG 100 spectrophotometer (Nano Drop Technologies, USA).

**PCR assay** **detection of *Brucella spp* using PCR assay:** The extracted DNA (n=238) was used to perform the PCR assay; the PCR reactions was targeting a 325 bp region within the IS711 gene using a pair of species-specific primers [13]. PCR reaction was completed in a LifePro Thermal Cycler- (Bioer Technology, China), by the following cycling conditions: denaturation at 94°C for 5 min followed by 30 cycles of 93 °C for 45 sec, 69°C for 1 min, and 72°C for 1 min, finalized by a final extension at 72 °C for 5 min.

**Detection of** ***B. abortus*** **and** ***B. melitensis*** **species using PCR** **: **The detection of *B. abortus* and *B. melitensis* species was performed using primers presented by Brocker and Halling [14]. The positive control was donated by Department of Pathobiology, School of Veterinary Medicine, Shiraz University, Iran. The following conditions was then applied; denaturation at 94°C for 5 min and then 32 cycles of 94 °C for 50 sec, 58 °C for 1 min, and 72 °C for 45 sec, followed by a final extension at 72°C for 5 min. In two phases of PCR; a 25 μL volume of reaction mixture of was containing 12.5 μL of master mix (Amplicon, Fermentas, Tehran, Iran), 1 μM of each primer, 150 ng of DNA preparation and 8.5 μL of nuclease-free water. Positive control and negative control (Ultra -pure Water DNase and RNase-free, Cinnagen, Tehran, Iran) were also incorporated in each assay. In the next step, 8 μL of PCR products were resolved by electrophoresis on a 1.5% (w/v) agarose gel was run onto a 1.5% of agarose gel stained with safe mode DNA stain (SinaClon, Tehran, Iran) and visualized by a UV transilluminator.

**Statistical analysis: **SPSS software (version 21; SPSS Inc., Chicago, IL, USA) was used to analyzed all the data. Prevalence of sample infection was computed with 95% confidence interval (CI).

## RESULTS

In this study, a total of 238 unpasteurized dairy products were tested for Brucella genus using a Nested PCR assay. Totally, 12 samples (5.04 %); (including 9 out of 48 raw milk samples (18.75 %), 3 out of 48 yogurt samples (6.25%) were reported to be positive (Table 1) which was finally confirmed by observing a 325 bp amplicon in the Nested PCR assay. Contamination was not detected in samples of dough, cheese and traditional ice cream.

**Table 1:** Frequency of *B. melitensis* and *B. abortus* in non-pasteurized dairy products samples

**Product type**	**Total number**	**Positive number**	**Positive number of B.** **abortus**	**Positive number of B.** **melitensis**	**Positive numbers of 1 abotus and B. melitensis simultaneously**	**Total Prevalence** **(95% CI)**
**Milk**	48	9	4	2	3	18.8 (4.8-29.1)
**Yogurt**	48	3	2	0	1	6.3 (0.0-14.6)
**Dough**	48	0	0	0	0	0
**cheese**	46	0	0	0	0	0
**Ice Cream**	48	0	0	0	0	0
**Total**	238	12	6	5	4	5 (2.5-8.0)

At this stage, the PCR assay was conducted using the relevant primers to amplify the *B. abortus* and *B. melitensis* specific genes. The results showed that among 12 positive samples (including 9 milk samples and 3 yogurt samples) in the previous stage, 6 samples were contaminated with *B. abortus* (including 4 milk samples and 2 yogurt samples), 2 samples were contaminated with *B. melitensis* (including 2 Milk samples) and 4 samples were contaminated simultaneously with both *B. abortus* and *B. melitensis* (including 3 milk samples and 1 yogurt sample) (Table 1). This was confirmed by observing a 498bp fragment for *B. abortus* and a 731 bp fragment for *B. melitensis *in the PCR assay.

## DISCUSSION

The health of milk and dairy products is very important because of their high nutritional value in the human nutrition [4]. Brucellosis as a zoonosis is still considered as one of the important public health concerns in many countries of the world including Iran [6]. Infected animals and dairy products, such as butter, fresh cheese and ice cream, are playing imperative roles in the transmission of disease in human [15]. Therefore, the consumption of milk and unpasteurized dairy products are very important regarding their serious and potential risk for the transmission of Brucella to humans [16]. According to the published data in the United States (1971-1978), 10% of the total of 1936 people affected with brucellosis mainly through the ingestion of unpasteurized milk and other dairy products [17].

The recent study showed that 12 samples (5.04%) out of 238 unpasteurized dairy products were reported positive for Brucella (including 9 raw milk samples, 3 yogurt samples). Contamination was not detected in samples of dough, cheese and traditional ice cream. The results also showed that among 12 positive samples, 6 samples were contaminated with *B. abortus*, 2 samples were contaminated with *B. melitensis* and 4 samples were contaminated simultaneously with *B. abortus* and *B. melitensis*.

 Several studies conducted in different regions of the world have focused on the rate of contamination of raw milk and dairy products with Brucella species. According to the study carried out by Miyashiro *et al*, among the 192 cheese samples, all of them were negative for *Brucella spp*. in the conventional culture, while 19.27% of the samples were found positive for in the PCR assay [18]. In Iraq, 8% of cheese and 1% of cream samples were respectively positive for B*. abortus* and *B. melitensis*, however, all samples of ice cream were reported to be negative [19]. According to a study conducted in Turkey in 2013, among 334 cow's milk samples, 273 samples (81.7%) were contaminated with *B. abortus *[20].

In addition, 110 milk samples from suspected cattle sheep were collected in different parts of Kurdistan province. *B. abortus* was identified in 9 samples (biovar 1, 2 and 4), and *B. melitensis* were detected in 2 samples of 20 cattle positive samples, using species-specific PCR assay, out of the 22 sheep positive samples, 15 samples were confirmed as *B. melitensis* and 1 sample was identified as *B. abortus* (biovar 1, 2 and 4). As a result, keeping animals in a close contact may interact with the host specificity [21].

In a similar study carried out in Isfahan and Chaharmahal and Bakhtiari Provinces, the contamination rate of the raw cow's milk (1% *B. abortus*), cheese (2.5% *B. abortus*, *B. mellitensis*), and cream (1% *B. abortus*) were reported among 200 samples , and no Brucella contamination was reported in the ice cream [16].

The most important reasons regarding the difference in the prevalence of Brucella species in dairy products consumed in different parts of the world can be attributed to the diversity of the geographical area, the type of survey, the sensitivity of the tests, the type and number of samples taken, the host factors, the vaccination of Brucella in animals and the methods used for the production of dairy products [22]. Since the presence of Brucella species in milk and unpasteurized dairy products has been proven in this study and sufficient heating can eliminate these microorganisms, pasteurization of milk and other dairy products can effectively prevent this disease in humans.

## References

[1] Young EJ, Corbel MJ (1989). Brucellosis: clinical and laboratory aspects.

[2] Gwida M, Al Dahouk S, Melzer F, Rösler U, Neubauer H, Tomaso H (2010). Brucellosis–regionally emerging zoonotic disease?. Croat Med J.

[3] Masoudian M, Derakhshandeh A, Ghahramani Seno MM (2015). Brucella melitensis and Mycobacterium tuberculosis depict overlapping gene expression patterns induced in infected THP-1 macrophages. Iran J Vet Res.

[4] Young EJ (1995). An overview of human brucellosis. Clin Infec Dis.

[5] Pappas G, Akritidis N, Bosilkovski M, Tsianos E (2005). Brucellosis. The New Eng J Med.

[6] Esmaeili H (2014). Brucellosis in Islamic republic of Iran. J Med Bacteriol.

[7] Jawetz E, Melnick JL, Adelberg EA (1983). Review of medical microbiology. The Pediat Inf Dis J.

[8] Corbel MJ (2006). Brucellosis in humans and animals: World Health Organization.

[9] Spanu V, Spanu C, Virdis S, Cossu F, Scarano C, De Santis EPL (2012). Virulence factors and genetic variability of Staphylococcus aureus strains isolated from raw sheep's milk cheese. Int J Food Microbiol.

[10] Izadi A, Moslemi E, Tabatabaei Panah A, Kheiri Manjili H (2014). Brucella spp. detection in dairy products using nested and hemi nested PCR techniques. Ann Biol Res.

[11] Bricker BJ (2002). PCR as a diagnostic tool for brucellosis. Vet Microbiol.

[12] Gupta VK, Verma DK, Rout PK, Singh SV, Vihan VS (2006). Polymerase chain reaction (PCR) for detection of Brucella melitensis in goat milk. Small Rum Res.

[13] Al Nakkas AF, Wright SG, Mustafa AS, Wilson S (2002). Single-tube, nested PCR for the diagnosis of human brucellosis in Kuwait. Ann Trop Med Parasitol.

[14] Bricker BJ, Halling SM (1994). Differentiation of Brucella abortus bv 1 2 and 4 Brucella melitensis, Brucella ovis and Brucella suis bv 1 by PCR. J Clin Microbiol.

[15] Doganay M, Aygen B (2003). Human brucellosis: an overview. Int J Infect Dis.

[16] Shakerian A (2015). Study of contamination rate in craw milk and its traditional products with Brucella abortus and Brucella mellitensis in Isfahan and Chaharmahal and Bakhtiari Provinces 2012. J Shahrekord Univ Med Sci.

[17] Bryan FL (1983). Epidemiology of milk-borne diseases. J Food Prot.

[18] Miyashiro S, Scarcelli E, Piatti RM, Campos FR, Vialta A, Keid LB, Dias RA, Genoves ME (2007). Detection of Brucella abortus DNA in illegal cheese from São Paulo and Minas Gerais and differentiation of B19 vaccinal strain by means of the polymerase chain reaction (PCR). Braz J Microbiol.

[19] Talei BAAAB (2010). Isolation, identification and biotyping of Brucella spp from milk product at Basrah Province. Basrah J Vet Res.

[20] Arasoglu T, Gulluce M, Özkan H, Adiguzel A, Şahin F (2013). PCR detection of Brucella abortus in cow milk samples collected from Erzurum, Turkey. Turk J Med Sci.

[21] Shafei B, Ahmadi M, Dastmalchi SH (2013). Diagnosis of Brucella abortus and Brucella melitensis in the milk of cattle and sheep in Kordestan province by polymerase chain reaction.

[22] Esmaeili H, Ekhtiyar ZH, Ebrahimzadeh H, Partovi R, Marhamati KB, Hamedi M, Khaji L (2012). Evaluation of the national sheep and goat brucellosis control program in Iran. Arak Uni Med Sci.

